# Factors influencing the efficiency of generating genetically engineered pigs by nuclear transfer: multi-factorial analysis of a large data set

**DOI:** 10.1186/1472-6750-13-43

**Published:** 2013-05-20

**Authors:** Mayuko Kurome, Ludwig Geistlinger, Barbara Kessler, Valeri Zakhartchenko, Nikolai Klymiuk, Annegret Wuensch, Anne Richter, Andrea Baehr, Katrin Kraehe, Katinka Burkhardt, Krzysztof Flisikowski, Tatiana Flisikowska, Claudia Merkl, Martina Landmann, Marina Durkovic, Alexander Tschukes, Simone Kraner, Dirk Schindelhauer, Tobias Petri, Alexander Kind, Hiroshi Nagashima, Angelika Schnieke, Ralf Zimmer, Eckhard Wolf

**Affiliations:** 1 Molecular Animal Breeding and Biotechnology, and Laboratory for Functional Genome Analysis (LAFUGA), Gene Center, LMU Munich, Munich, Germany; 2 Practical Informatics and Bioinformatics, Institute for Informatics, LMU Munich, Munich, Germany; 3 Livestock Biotechnology, Center of Life and Food Sciences Weihenstephan, TU Munich, Freising, Germany; 4 International Institute for Bio-Resource Research, Meiji University, Kawasaki, Japan

**Keywords:** Transgenic pig, Knockout pig, Somatic cell nuclear transfer, Multi-factorial analysis

## Abstract

**Background:**

Somatic cell nuclear transfer (SCNT) using genetically engineered donor cells is currently the most widely used strategy to generate tailored pig models for biomedical research. Although this approach facilitates a similar spectrum of genetic modifications as in rodent models, the outcome in terms of live cloned piglets is quite variable. In this study, we aimed at a comprehensive analysis of environmental and experimental factors that are substantially influencing the efficiency of generating genetically engineered pigs. Based on a considerably large data set from 274 SCNT experiments (in total 18,649 reconstructed embryos transferred into 193 recipients), performed over a period of three years, we assessed the relative contribution of season, type of genetic modification, donor cell source, number of cloning rounds, and pre-selection of cloned embryos for early development to the cloning efficiency.

**Results:**

109 (56%) recipients became pregnant and 85 (78%) of them gave birth to offspring. Out of 318 cloned piglets, 243 (76%) were alive, but only 97 (40%) were clinically healthy and showed normal development. The proportion of stillborn piglets was 24% (75/318), and another 31% (100/318) of the cloned piglets died soon after birth. The overall cloning efficiency, defined as the number of offspring born per SCNT embryos transferred, including only recipients that delivered, was 3.95%. SCNT experiments performed during winter using fetal fibroblasts or kidney cells after additive gene transfer resulted in the highest number of live and healthy offspring, while two or more rounds of cloning and nuclear transfer experiments performed during summer decreased the number of healthy offspring.

**Conclusion:**

Although the effects of individual factors may be different between various laboratories, our results and analysis strategy will help to identify and optimize the factors, which are most critical to cloning success in programs aiming at the generation of genetically engineered pig models.

## Background

Somatic cell nuclear transfer (SCNT) has become widely used for the generation of genetically engineered large animals, especially since germ line competent pluripotent stem cells – the key to sophisticated reverse genetics in rodents – are not available in these species [1-4]. Genetic modification of pigs by SCNT facilitated gene targeting [5-7], inducible transgene expression [8], and the first successful examples of zinc finger nuclease mediated targeted gene modifications [9,10] to generate tailored large animal models and donor animals for xenotransplantation.

During the last decade, transgenic pigs have gained importance in the field of biomedical research because of major anatomical and physiological similarities with humans [11] as well as the need for non-rodent based studies to investigate disease mechanisms, the efficacy and safety of new therapies, and to identify biomarkers for companion diagnostics. Genetically tailored pig models have already been developed to investigate cystic fibrosis [12], diabetes mellitus [13-16], and neurodegenerative diseases [17] (reviewed in [18]). Multiple lines of genetically modified pigs have also been generated for xenotransplantation (reviewed in [19]), most notably α1,3-galactosyl transferase knockout pigs lacking α1,3-Gal, the major xeno-antigen [5]. SCNT has facilitated the generation of donor pigs carrying multi-transgene combinations designed to overcome immune rejection and to ensure functional compatibility between xenograft and recipient, e.g. regulation of blood coagulation.

Although the first successful SCNT experiments using cultured porcine cells were performed more than a decade ago [20-22], the efficiency of cloning (live offspring per reconstructed embryos transferred to recipients) is still low, usually ranging from 1 to 5%, and cloned animals may suffer from various developmental defects.

Genetic modification of nuclear donor cells necessarily involves a series of procedures, such as transfection or transduction, drug selection and extended growth in culture, which could possibly affect their ability to support normal development.

To date, several studies have reported key factors in the production of cloned pigs and suggested a number of approaches to improve efficiency. However, the majority of these studies have addressed only single factors, e.g. SCNT procedure [23-26], oocyte and embryo culture systems [27,28], donor cell type [29,30], and the method of genetic modification [31,32]. Combined assessment of multiple factors and comparative analysis of their relative contribution to cloning efficiency have not yet been performed to our knowledge.

Here, we investigate the impact of five factors on the crucial stages of a cloning experiment and ultimately the impact on cloning efficiency. We used a large data set comprising three years of porcine SCNT experiments, during which more than 300 cloned pigs were generated using different genetically modified cell cultures. The data contains simultaneous variations in season, type of genetic modification (additive gene transfer vs. gene targeting), donor cell source (mesenchymal stem cells, postnatal fibroblasts, fetal fibroblasts, and kidney cells), number of cloning rounds, and pre-selection of cloned embryos for early development. We assessed the impact pattern of the variable factors on pregnancy and delivery rates as well as the numbers of born, live and healthy offspring. Cloning efficiency was calculated as the number of cloned piglets born relative to the number of SCNT embryos transferred to recipients that gave birth.

## Results

### General information

A total of 18,649 SCNT embryos were transferred into 193 recipients. The average number of embryos transferred per recipient was 97 (range: 43–216). 109 recipients (56%) became pregnant and 85 (78%) of those gave birth to offspring. The pregnancy rate was significantly increased when more than 100 NT embryos were transferred to a recipient. Experiments in which over 135 NT embryos were transferred resulted in the maximum overall pregnancy rate of 79.3% (Additional file 1). Recipients that became pregnant displayed no tendency for delivering live offspring in dependence on the number of embryos transferred (Additional file 2). Of the 318 cloned piglets born, 243 (76%) were alive, but only 97 (40%) were clinically healthy, defined as the absence of any visible anatomical or physiological disturbance, and showed normal development. The proportion of stillborn piglets was 24% (75/318), and another 31% (100/318) of the cloned piglets died soon after birth. The major reason for early neonatal death within 2 weeks was underweight (<1000 g) and/or weakness of unknown causes, which was observed in several transgenic litters. In addition, we observed malformations such as oversized tongue (30 cases, 9.4%), cleft palate (2 cases, 0.6%) or *atresia ani* (1 case, 0.3%), abnormalities of the legs (6 cases, 1.9%), patent *urachus* (1 case, 0.3%) and umbilical hernia (6 cases, 1.9%). In 3 cases (0.9%), piglets showed contracted tendons in the forelegs, which improved with increasing body weight and did not affect survival. 39 piglets (12%) were lost for other reasons (killed by the mother or died from infection). The health status of the remaining 7 cloned piglets could not be estimated as they have been used for experiments immediately after birth. The overall cloning efficiency, defined as the number of offspring born per SCNT embryos transferred, including only recipients that delivered, was 3.95%. A detailed description of the data set is shown in Table 1.

**Table 1:** Data summary

	
Total no. of transferred SCNT embryos	18,649
Average no. of transferred embryos per recipient	97
Range of transferred embryos per recipient	43-216
No. of different cell sources used for SCNT	4^1^
Type of genetic modification	
Additive gene transfer (no. of constructs)	14^2^
Homologous recombination (no. of target genes)	6^3^
Total no. of recipient pigs	193
Pregnant recipients	109 (56%)
Delivering recipients	85 (78%)
Total no. of cloned offspring	318
Live cloned pigs	243 (76%)
Healthy cloned pigs	97 (40%)

^1^ Mesenchymal stem cells, postnatal fibroblasts, fetal fibroblasts, and kidney cells.

^2^ See Table 8 for details.

^3^ See Table 9 for details.

### Impact of individual factors

We assessed the influence on the cloning outcome of five factors: the season the embryo transfer (ET) was performed in, the type of genetic modification, the donor cell source, the number of cloning rounds, and selection of SCNT embryos for development before transfer to the recipient. The stratification and distribution of each varied factor is summarized in Table 2 (more details can be found in Methods, Additional file 3 and Additional file 4).

**Table 2:** Stratification and data distribution of the investigated experimental factors

**Factor**	**No. of embryo transfers (%)**	
**Season**^**1**^		
- Spring	39	(20.2)
- Summer	59	(30.6)
- Autumn	58	(30.0)
- Winter	37	(19.2)
**Type of genetic modification**^**2**^		
- de novo - AGT	57	(29.5)
- HR	48	(24.9)
- replication of transgenic pig	88	(45.6)
**Donor cell source**^**3**^		
- MSC	36	(18.7)
- PF	24	(12.4)
- FF	51	(26.4)
- KC	82	(42.5)
**Cloning rounds**		
- 1 time	110	(57.0)
- 2 times	62	(32.1)
- 3 times	21	(10.9)
**Selection of SCNT embryos for early development**^**4**^		
- no selection	45	(23.3)
- selection after 1 day	13	(6.7)
- selection after 2 days	15	(7.8)
- mixed selection	120	(62.2)

Additional file 3 and Additional file 4 show in more detail the distribution in season and embryo selection of specific SCNT configurations with respect to genetic modification, donor cell source and number of cloning rounds.

^1^ Embryo transfer date.

^2^ AGT: additive gene transfer, HR: homologous recombination.

^3^ Mesenchymal stem cells (MSC), postnatal fibroblasts (PF), fetal fibroblasts (FF), and kidney cells (KC).

^4^ No selection: all SCNT embryos transferred, selection for 1 day: 1-cell stage SCNT embryos transferred, selection for 2 days: 2-cell to 4-cell stage SCNT embryos transferred, mixed selection: mixed SCNT embryos transferred (no selection/1 day and 1 day/2 days).

#### Season

The seasonal influence on the assessed parameters is presented in Table 3. Spring was used as the reference category and statistically significant differences of results obtained in other seasons are indicated relative to the reference category. The oocyte maturation rate was highest in spring (77.1%), slightly lower in autumn (75.8%) and summer (74.4%), and significantly decreased in winter (71.3%; *p* < 0.05). Similarly, significantly fewer pregnancies were established in winter (1:2 chance) than in spring (2:1 chance). In contrast, the proportion of offspring per SCNT embryos transferred (cloning efficiency) was highest when ET was performed in winter (5.3%), as compared to spring (3.5%; *p* < 0.05). Similarly, the average number of live cloned offspring from ET performed during winter (4.3) was significantly higher than during spring (2.6; *p* < 0.05). The lowest number of healthy cloned piglets was observed if the ET was done in summer (0.8 vs. 2.2 when ET was performed in winter).

**Table 3:** Seasonal variation pattern of the cloning outcome

**Season**	**Temperature (°C)**^**1**^	**Oocyte maturation (%)**	**Chance for pregnancy**	**Chance for delivery**	**Cloning efficiency (%)**	**No. of live cloned piglets**	**No. of healthy cloned piglets**
Spring	9.6	77.1	2	4.2	3.5	2.6	1.4
Summer	18.1	74.4	1.2	3.6	3.8	3.0	0.8*
Autumn	9.2	75.8	1.8	3.1	4.0	2.6	1.4
Winter	0.1	71.3*	0.6*	3.7	5.3*	4.3*	2.2

Spring was used as the reference category. For details on the statistical analysis and definition of the cloning benchmarks listed, please see Methods.

* Statistically significant differences (*p* < 0.05).

^1^ Average temperature in Munich during the experiments.

#### Type of genetic modification

Genetic modifications were categorized into three classes: additive gene transfer, homologous recombination, and replication of already existing transgenic pigs. The effects of these classes of genetic modification on outcome are summarized in Table 4. Homologous recombination was used as the reference category. No significant difference was apparent between these three classes of modification with regard to cloning efficiency, pregnancy and delivery rate. However, the numbers of live and healthy cloned offspring per litter, respectively, were significantly higher (*p* < 0.05) in the additive gene transfer group than in the homologous recombination group (3.5 vs. 2.3 and 1.5 vs. 0.6, respectively).

**Table 4:** Variation of the cloning outcome depending on the type of genetic modification

**Genetic modification**^**1**^	**Chance for pregnancy**	**Chance for delivery**	**Cloning efficiency (%)**	**No. of live cloned piglets**	**No. of healthy cloned piglets**
HR	1	4	3.8	2.3	0.6
AGT	1.8	6.2	4.2	3.5*	1.5*
Replication of transgenic pigs	1	2.4	3.9	2.7	1.2

Homologous recombination (HR) was used as the reference category. For details on the statistical analysis and definition of the cloning benchmarks listed, please see Methods.

* Statistically significant differences (*p* < 0.05).

^1^*HR*: homologous recombination, *AGT*: additive gene transfer, Replication of transgenic pigs: replication of already existing transgenic pig lines.

#### Nuclear donor cell source

Four different cell sources – mesenchymal stem cells, fetal fibroblasts, postnatal fibroblasts, and kidney cells – were used and their effect on cloning success was determined (Table 5). Mesenchymal stem cells were used as the reference category. The fusion rate of mesenchymal stem cells (93%) was significantly (*p* < 0.05) higher, while that of postnatal fibroblasts (80%) was lower than those of other donor cells. The pregnancy rate was highest with fetal fibroblasts, and lowest with postnatal fibroblasts used as donor cells, but the differences between donor cell sources were not statistically significant. In contrast, the delivery rate was higher with mesenchymal stem cells than with fetal fibroblasts and kidney cells. The cloning efficiency was not affected by the source of donor cells. The proportion of live and healthy cloned offspring in the fetal fibroblast and kidney cell groups was higher than in the mesenchymal stem cell reference group.

**Table 5:** Variation of the cloning outcome depending on different nuclear donor cell sources

**Cell source**^**1**^	**Fusion rate (%)**	**Chance for pregnancy**	**Chance for delivery**	**Cloning efficiency (%)**	**No. of live cloned piglets**	**No. of healthy cloned piglets**
MSC	93.0	1.1	5.3	3.5	1.6	0.3
PF	80.2*	0.7	4.0	4.1	2.0	0.5
FF	89.1*	1.8	3.7*	4.4	3.4*	1.9*
KC	90.4*	1.3	2.9*	3.8	3.4*	1.4*

Mesenchymal stem cells (MSC) were used as the reference category. For details on the statistical analysis and definition of the cloning benchmarks listed, please see Methods.

* Statistically significant differences (*p* < 0.05).

^1^ Mesenchymal stem cells (*MSC*), postnatal fibroblasts (*PF*), fetal fibroblasts (*FF*), and kidney cells (*KC*).

#### Number of cloning rounds

In this data set, up to three rounds of nuclear transfer were performed. One cloning round was used as the reference category (Table 6). Although no statistically significant difference was apparent in pregnancy and delivery rates, cloning efficiency decreased significantly (*p* < 0.05) with cloning round (4.4%, 3.5% and 2.9% for one, two and three cloning rounds, respectively). The number of live and healthy offspring after two rounds was significantly (*p* < 0.05) lower than after the first cloning round (2.2 vs. 3.2 and 0.5 vs. 1.7, respectively). This effect was not seen after three rounds of SCNT.

**Table 6:** Influence of the number of cloning rounds on the cloning outcome

**No. of cloning rounds**	**Chance for pregnancy**	**Chance for delivery**	**Cloning efficiency (%)**	**No. of live cloned piglets**	**No. of healthy cloned piglets**
1	1.3	3.4	4.4	3.2	1.7
2	1.0	6.8	3.5*	2.2*	0.5*
3	3.2	1.7	2.9*	3.1	1.6

One cloning round was used as the reference category. For details on the statistical analysis and definition of the cloning benchmarks listed, please see Methods.* Statistically significant differences (*p* < 0.05).

#### Selection of cloned embryos for initiation of development

The effect of selection of SCNT embryos on the cloning outcome is shown in Table 7. As reference category, we used the cases where no selection was performed. Pregnancy and delivery rates were not significantly affected by *in vitro* culture of cloned embryos and selection for early development. However, transfer of *in vitro* cultured SCNT embryos, which had developed to 2-cell to 4-cell stage, resulted in the highest proportion of offspring per embryos transferred (6.8% vs. 4.5% in the group where no selection was performed; *p* < 0.05). The numbers of live and healthy offspring were not affected by the pre-selection of cloned embryos for early development.

**Table 7:** Effect of SCNT embryo selection on the cloning outcome

**Selection timing**^**1**^	**Chance for pregnancy**	**Chance for delivery**	**Cloning efficiency (%)**	**No. of live cloned piglets**	**No. of healthy cloned piglets**
No selection	1.1	1.9	4.5	3.4	1.4
Selection for 1 day	0.9	-	4.9	4.3	1.5
Selection for 2 days	0.6	4.0	6.8*	3.2	2.0
Mixed selection	1.8	4.0	3.5	2.6	1.3

No selection was used as the reference category. For details on the statistical analysis and definition of the cloning benchmarks listed, please see Methods.

* Statistically significant differences (*p* < 0.05).

^1^ No selection: all SCNT embryos transferred; selection for 1 day: 1-cell stage SCNT embryos transferred; selection for 2 days: 2-cell to 4-cell stage SCNT embryos transferred; mixed selection: mixed SCNT embryos transferred (no selection/1 day and 1 day/2 days)

### Statistically significant effects on different phases of development

#### From *in vitro* oocyte maturation to cloned offspring

**Figure 1:**
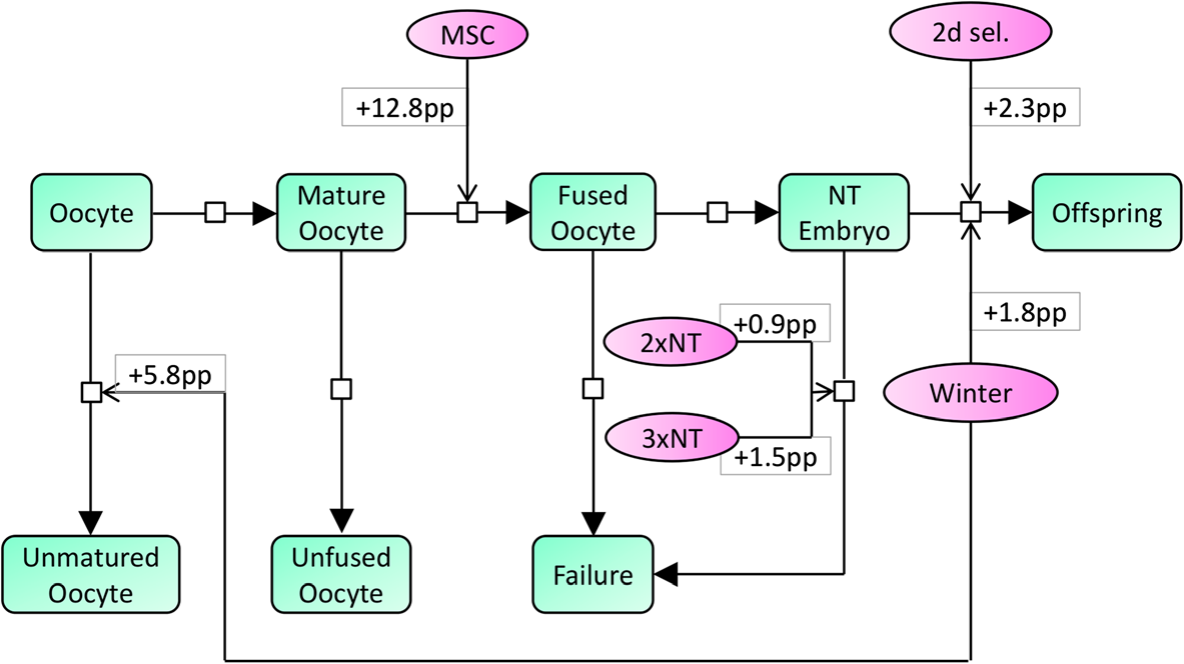
**The major statistical effects of the investigated factors on the development from *in vitro *oocyte maturation to cloned offspring.** According to the results listed in Tables 3, 4, 5, 6, and 7, transitions between developmental stages (green rectangles) are found to be affected by statistically significant impact categories (purple ellipses). For each impact, the gain during a certain transition is given in percentage points (pp) and as compared to the corresponding reference. For example, the cloning efficiency (offspring out of transferred embryos, including only recipients that delivered) is increased by 1.8 pp in winter (from 3.5% to 5.3%), as compared to spring, which was used as reference. MSC: mesenchymal stem cells, 2d sel.: selected embryos for initiation of development on day 2 (2-cell to 4-cell stage), NT: nuclear transfer.

As shown in Figure 1, the maturation of oocytes was significantly impaired in winter (reduced by almost 6 percentage points as compared to spring). We found high fusion rates to be associated with the use of mesenchymal stem cells (up to 13 percentage points better than other cell sources). Cloning efficiency and, thus, the chance for full term development was improved when 2-cell to 4-cell embryos, selected after 2 days *in vitro* culture, were transferred to the recipient. In contrast, the cloning efficiency was negatively affected by repeated SCNT (two rounds of cloning).

#### Outcome stage of the recipients (pregnancy/delivery) and the offspring (live/healthy)

**Figure 2:**
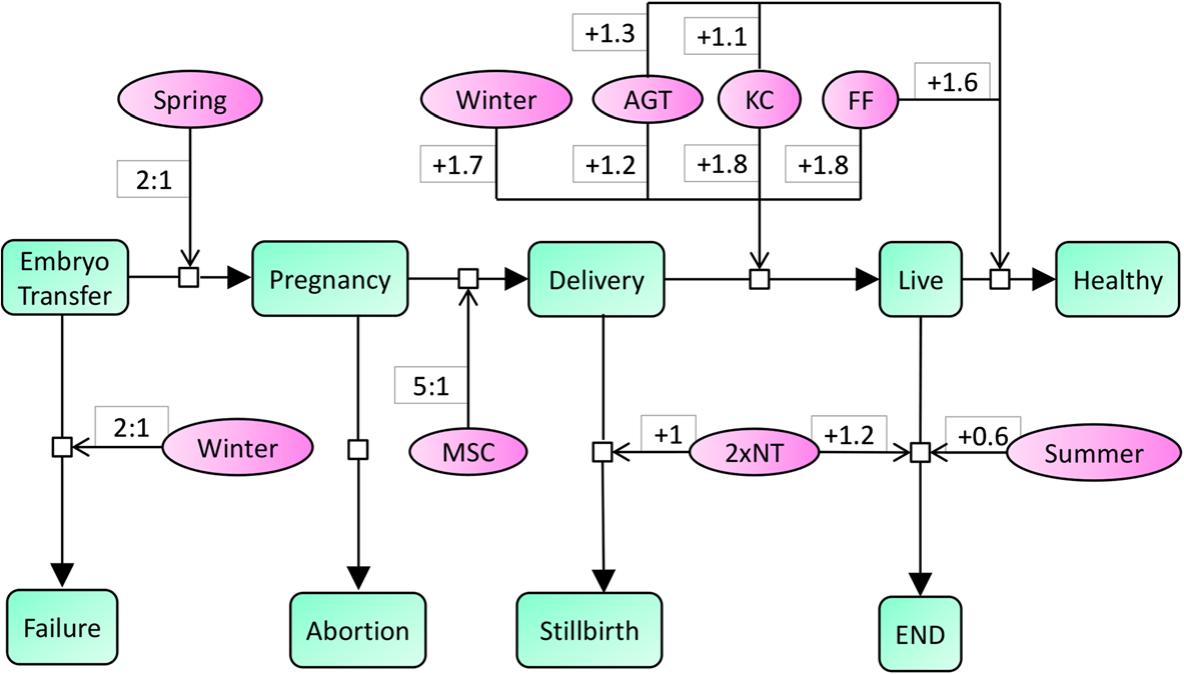
**The major statistical effects of the investigated factors on the outcome stage of the recipients (pregnancy/delivery) and the offspring (live/healthy).** For each impact, the gain during a certain transition is given in the respective outcome unit. For example, there are on average 1.7 more alive piglets for cloning in winter, as compared to the reference (cloning in spring). MSC: mesenchymal stem cells, AGT: additive gene transfer, KC: kidney cells, FF: fetal fibroblasts, NT: nuclear transfer, END: early neonatal death.

As illustrated in Figure 2, we frequently observed pregnant pigs in spring (chance for pregnancy 2:1, i.e. the probability *P* (pregnancy = YES) was twice as high as the probability *P* (pregnancy = NO)), whereas pigs hardly became pregnant in winter (1:2 chance). In addition, delivering recipients occurred mostly when mesenchymal stem cells were used (superior chance for delivery of 5:1). Most noteworthy, two cloning rounds significantly (*p* < 0.05) increased the risk of both, pre- and post-natal death of cloned fetuses and offspring, respectively. Post-natal death of offspring also frequently occurred when the embryo transfer was performed in summer and the piglets were therefore born in late autumn/early winter. On the other hand, statistically significant increases in the offspring outcome were observed for embryo transfer in winter, genetic modification by additive gene transfer, and the use of fetal fibroblasts or kidney cells as nuclear donors. These experimental settings yielded on average between one and two more live and healthy piglets than the corresponding reference category.

Interestingly, we could confirm beneficial effects of cloning in winter, using additive gene transfer, and fetal fibroblasts or kidney cells, in an additional analysis (data not shown), where we explicitly targeted the fraction of early neonatal death cases out of live piglets in experiments, which in principle could produce viable offspring (indicated by at least one live piglet). Cloning in winter, using additive gene transfer, and fetal fibroblasts or kidney cells, resulted in 12 percentage points, 32 percentage points, and >35 percentage points less early neonatal death cases, as compared to the reference categories cloning in spring, using homologous recombination, and mesenchymal stem cells, respectively.

## Discussion

The outcome of somatic cell nuclear transfer is affected by complex interactions between multiple factors. While some of these are difficult to control, others – such as choice of nuclear donor cell source – may help increase the efficiency of cloning.

Over a period of three years, we generated more than 300 genetically modified pigs by SCNT using multiple donor cell sources. These cells were either *de novo* modified by additive gene transfer or gene targeting, or were derived from existing transgenic or knockout pig lines. All data were collected within our routine workflow for the production of genetically engineered pigs for biomedical research [18]. We used this large data set to identify factors that affect efficiency of cloning and at which stage they act.

We have employed robust linear models, requiring minimal distribution assumptions adjusted to the underlying empirical distribution of the cloning outcome, as a straightforward approach to determine the statistically significant part of the network of factors affecting pig cloning. As shown in Figures 1 and 2, network-based interpretation concepts were used to model and discriminate the major genetic, environmental and experimental factors.

The factors addressed by our study influenced the outcome of cloning for the production of genetically modified pigs on different levels.

The season affected *in vitro* maturation of oocytes, pregnancy rate, and survival of cloned piglets. Even if the domestic pig shows an estrus cycle with fertility throughout the year, the reproductive performance in commercial pig breeding is notably reduced in late summer and early autumn [33-35]. Bertoldo et al. [36] have documented reduced developmental competence of oocytes during this period. In our data set, the best maturation rate of oocytes *in vitro* was observed in spring and the worst in winter. The latter may be caused, at least in part, by accidental exposure of oocytes to low temperatures during collection and transport. Pig oocytes are very sensitive to low temperature due to high levels of cytoplasmic lipids [37]. During the time span between removal from the incubator and finished embryo transfer, maintenance of an optimal temperature cannot always been guaranteed. Therefore, low temperatures might affect the developmental capability of the embryos and could be responsible for the lower pregnancy rate after ETs in winter, compared to ETs in spring. Nevertheless, cloning efficiency was highest when SCNT experiments and ETs were performed in winter. This finding seems to be contradictory on a first view, but it has to be considered that the cloning efficiency was calculated only for cases in which the recipient delivered offspring. Cases of unsuccessful transfers were not included into the calculation. Therefore, if the embryos survived the negative environmental influences in winter and the recipients became pregnant, the natural high fertility period of the recipients might provide a favorable environment for embryos and fetuses to develop to term.

Unexpectedly, the method of genetic modification had little effect on cloning efficiency in our data set. Generally, genetic modification of donor cells requires prolonged *in vitro* culture for transfection and selection, which could induce cellular changes leading to a decrease in cloning efficiency. Gene targeting by homologous recombination takes a particularly long time and multiple cell divisions to establish single cell clones with sufficient cell numbers for genetic analysis and nuclear transfer [5,38-40]. In contrast, our protocol for additive gene transfer uses pools of mixed cell clones, which have been maintained under selection for 7 to 10 days [8,18,41]. It might therefore be expected that extended *in vitro* culture of donor cells required for homologous recombination would negatively influence the cloning efficiency, compared to cells modified by additive gene transfer. However, our data showed no statistically significant difference in cloning efficiency between additive gene transfer, homologous recombination or replication of already existing transgenic pigs. It can be hypothesized that the conditions for transfection and selection did not adversely affect the developmental potential of donor cells, since we kept the passage numbers for SCNT donor cells as low as possible - less than 8 passages for additive gene transfer and less than 10 passages for gene targeting. Additionally, all wild-type primary cell lines used in this study were karyotyped and showed 68% to 90% normal karyotypes.

Interestingly, our analysis indicated that the number of live and healthy offspring was decreased when nuclear donor cells had undergone homologous recombination. However, this may – at least in part – be explained by the fact that 65% of nuclear transfers, designed to generate gene-targeted pigs, were carried out using only 4 particular mesenchymal stem cell lines, which later on turned out to be consistently poor in producing live cloned offspring.

Another important aspect to be considered in the context of genetic modification is the potential for lethal or toxic effects of modifications *per se*. For the experiments involving additive gene transfer this is unlikely, since live cloned piglets expressing the transgenes were obtained with all constructs. Nevertheless, we cannot rule out that cloned fetuses or offspring died due to a detrimental random integration of the construct. Of the gene targeting experiments, only mutation of the X-linked dystrophin (*DMD*) gene in male clones may cause a severe phenotype. In fact, *DMD* mutant male piglets showed severe muscular dystrophy already at birth, and a proportion died shortly later [42]. For all other target genes, the heterozygous knockout had either no specific phenotype or a phenotype that develops later in life.

A critical factor for the establishment of genetically engineered pig lines by SCNT is the viability of the cloned founder animals up to sexual maturity. In our data set, more than half of cloned pigs were stillborn (23.6%) or died soon after birth (31.4%). Associated pathological changes, such as underweight (average weight of the cloned piglets born under 1000 g: 686.4 +/- 181.0 g; range: 375 – 973 g), which is one of the major causes of early neonatal death, or cleft palate, contracted tendons, or enlarged tongues, have also been observed by other groups [43-47]. We have the impression that the percentage of underweight piglets (among normal weight littermates) is higher in cloned litters. However, we cannot prove this observation by statistical data, as the birth weights of naturally bred piglets are not routinely recorded in our facility. The average birth weight of healthy cloned pigs was higher than that of piglets that died in the neonatal period, or that of stillborn piglets (1409.2 +/- 343.1 g, 974.8 +/- 394.1 g, and 1065.5 +/- 479.0 g, respectively). These abnormalities could not be associated with any particular parameter, like donor cell source or genetic modification, and might be a general side effect in pig cloning. Previous studies reported that phenotypically abnormal cloned animals could produce normal offspring [48,49], suggesting that phenotypic abnormalities of the clones were more likely due to epigenetic rather than to genetic alterations.

In our data set, cloned piglets with enlarged tongues were mainly observed in offspring cloned from bone marrow derived mesenchymal stem cells, originating from 4 different animals (25 of 30 cases). However, this does not seem to be a general feature of mesenchymal stem cells, since in more recent cloning experiments with adipose tissue derived mesenchymal stem cells a high proportion of viable offspring without malformations was obtained (T. Flisikowska and A. Schnieke, unpublished data). Some groups reported that mesenchymal stem cells are superior to fibroblasts for SCNT in pigs [50-53], although this has not been generally observed [40,54,55]. Our results did not show any differences in the cloning efficiency among the different cell sources tested, although there was a tendency for a higher pregnancy rate when mesenchymal stem cells were used. The observation that the numbers of live and healthy cloned piglets were significantly lower in the mesenchymal stem cells group than other donor cell sources may be due to the fact that mesenchymal stem cells were only used for gene targeting. Thus, it cannot be distinguished at this stage, whether the low outcome of live and healthy piglets can be attributed to the cell source or type of genetic modification. In addition, different cell lines derived from the same cell source showed a considerable degree of variation in cloning efficiency (Additional file 5).

Re-cloning by using cells from a cloned animal for NT is a reasonable approach for the reproduction of specific transgenic animals, for example if animals of a defined genotype are required for an experiment or if the phenotype hinders natural breeding. However, the majority of studies on re-cloning have demonstrated that additional rounds of cloning lead to a decrease in cloning efficiency [49,56-58]. Our data also showed that repeated cloning rounds significantly decreased cloning efficiency (R1: 4.4%, R2: 3.5% and R3: 2.9%), and the number of live cloned offspring in the second round was in average one piglet less as compared to the initial cloning round. It should be mentioned that the lowest cloning efficiency for R3 may also be related to the high number of embryos transferred in these experiments. Xing et al. [59] recently demonstrated that reduced developmental potential of pig embryos generated by multiple rounds of cloning was associated with altered gene expression patterns, and a previous report stated that the reduction of cloning efficiency with additional rounds of cloning may be caused by accumulation of epigenetic errors [60].

The last factor addressed by our study was *in vitro* culture of cloned embryos and selection for normal development before transfer to recipients. This is possible since the *in vitro* culture systems for pig embryos have been markedly improved within the last decade [27,28]. Indeed, culture of embryos for two days and selection of 2-cell to 4-cell stage embryos for ET resulted in the highest proportion of offspring per SCNT embryos transferred. This suggests that SCNT embryos, which undergo normal cleavage *in vitro* within the expected time frame, have a greater chance of full term development *in vivo*.

## Conclusion

We have investigated the influence of important experimental and environmental factors on the cloning outcome in a considerably large data set comprising over 270 porcine nuclear transfer experiments. Besides assessment of the cloning efficiency, we determined the respective steps of the cloning process from oocyte to offspring that are most critically influenced. We observed varying effects of individual factors, depending on the combination with other chosen factors and the parameters tested. Most importantly, more live and healthy offspring were obtained when fetal fibroblasts or kidney cells were modified by additive gene transfer and the resulting SCNT embryos were transferred in the winter period. Although our results cannot be simply extrapolated to other cloning labs, the approach used in this study may help to identify and optimize the specific factors most critical to cloning success in programs aiming to generate genetically engineered pigs.

## Methods

### Ethics statement

All animal procedures in this study were performed according to the German Animal Welfare Act and to a protocol approved by the Regierung von Oberbayern, under the reference numbers (55.2.1.54-2531-26-06; 55.2.1.54-2531-77-07; 55.2.1.54-2531-78-07; 55.2.1.54-2531-136-07; 55.2.1.54-2531-54-08; 55.2.1.54-2531-86-10; 55.2.1.54-2532-68-11).

### Generation of genetically modified pigs

Genetically modified cells derived by transfection of primary cells or established from already existing transgenic pig lines were used as donors. The cells derived from transfection were genetically modified by additive gene transfer (Table 8) or by homologous recombination (Table 9). The latter group included bacterial artificial chromosome (BAC) targeting [7] and the use of classical targeting vectors [5]. The cells re-established from already existing transgenic pig lines were collected from 18 different transgenic pigs. Individual information on all cell lines used for these analyses is shown in the Additional file 6 and Additional file 7.

**Table 8:** Gene constructs for additive gene transfer

**Gene**	**Promoter**	**Coding sequence**	**3’-UTR/pA**
hTM	8.9 kb poTM	1.9 kb huTM gene	0.3 kb boGH
CAG-Case12	1.7 kb CAG [61]^§^	1.2 kb Case12^a^	0.6 kb raHBB [61]^§^
CAG-TA [8]^§^	1.7 kb CAG [61]^§^	1.0 kb rtTA^b^	0.3 kb boGH
CAG-LEA	1.7 kb CAG [61]^§^	1.2 kb LEA29Y^c^	0.6 kb raHBB [61]^§^
INS-LEA [62]^§^	1.5 kb po INS	1.2 kb LEA29Y^c^	0.3 kb boGH
INS-C94Y [16]^§^	2.5 kb po INS fragment including point mutation		
INS-C93S	2.5 kb po INS fragment including point mutation		
INS-TK	1.5 kb po INS	1.1 kb TK^c^	0.3 kb boGH
COL-TK	3.6 kb po COL1A1	1.1 kb TK^c^	0.3 kb boGH
CFTR-LacZ	CH242-248P18	3.5 kb lacZ^d^	0.3 kb boGH
GGTA-LacZ	CH242-21 F3	3.5 kb lacZ^d^	0.3 kb boGH
TRE-RANKL [8]^§^	0.3 kb TRE^a^	0.9 kb po sRANKL	0.3 kb boGH
TRE-CTLA-4Ig [8]^§^	0.3 kb TRE^a^	1.2 kb po CTLA4-Ig	0.3 kb boGH
HAC1 [63]^§^	0.6 kb CMV	0.7 kb GFP	0.3 kb SV40

^§^ See indicated references.

^a^ Purchased from Evrogen, Moscow, Russia.

^b^ Purchased from Clontech, Mountain View, CA.

^c^ Custom-synthesized by Bio&Sell, Feucht, Germany.

^d^ Purchased from Promega, Madison, WI.

**Table 9:** Target genes for homologous recombination

**Target gene**	**Vector**	**Modification**
*CFTR*[7]^§^	CH242-248P18 (>100 kb)	ATG-STOP
*DMD*[42]^§^	CH242-9G11 (>100 kb)	Δ exon 52
*GGTA1*	CH242-21 F3 (>100 kb)	ATG-STOP
*APC*[64]^§^	12.5 kb	STOP
*KRAS*	13.5 kb	Point mutation
*JAK3*	13.8 kb	Δ exon 2-5

^§^ See indicated references.

The following cell sources were used: mesenchymal stem cells, postnatal fibroblasts, fetal fibroblasts, and kidney cells. Mesenchymal stem cells, multi-potent tissue stem cells, as well as fibroblasts and kidney cells are already known to support full term development when used as donor cells in pig SCNT [21,23,41,53,64-67]. Briefly, mesenchymal stem cells from bone marrow were isolated from femurs and tibias of 6 to 7 months old Landrace x Pietrain pigs [64,68]. Fetal fibroblasts, postnatal fibroblasts and kidney cells originated from German Landrace, Swabian-Hall pig and crossbreeds of them [41]. Fetal fibroblasts were isolated from fetuses at day 27 and day 54, while postnatal fibroblast and kidney cells were from 1 day up to 3 months old piglets. The gender of all cell lines was male, except for one of the *GGTA1*^-/-^ CD46 cell lines, which was female. Donor cells were isolated by standard methods mainly using collagenase II or trypsin/EDTA [41]. For details of transfection and characterization of *de novo* modified cells see references [7,8,41,64]. Cells were used for SCNT at passage 6–8 after additive gene transfer, passage 6–10 after homologous recombination, and passage 2–8 from re-established transgenic pig lines. 48 h prior to the SCNT experiment, donor cells were starved (0.5% FCS) for synchronization of donor cell cycle at G0/G1. All SCNT experiments included in this analysis were performed in the same laboratory by the same operators for micro-manipulation, using *in vitro* matured (IVM) oocytes, as previously reported [67].

Up to three rounds of cloning (use of donor cells derived from an already cloned animal for a further round of SCNT) were performed for the generation and replication of multi-transgenic pigs. Specifically, one round of cloning was used for generating transgenic founder animals from transfected wild-type cells, or for replicating offspring of transgenic founder pigs. The second round of cloning involved donor cells from transgenic cloned pigs which were transfected with an additional construct or simply the replication of transgenic cloned pigs. In the third round, cloning was the re-cloning of transgenic pigs that had received an additional construct during the second round of cloning (for the individual information in each cell lines, see Additional file 6 and Additional file 7).

Fused reconstructed embryos were either directly transferred to recipients on the same day (no selection), or cultured *in vitro* and then selected for initiation of development on day 1 (1-cell stage) or day 2 (2-cell to 4-cell stage) after activation before embryo transfer.

Gilts of the breeds German Landrace, Swabian-Hall, and crossbreeds of them were used as recipients. Estrus was synchronized by oral administration of 4 ml Altrenogest (Regumate®) for 15 days, followed by intramuscular injection of 750 IU ECG (Intergonan®) and 750 IU HCG (Ovogest®) 24 h and 104 h later, respectively. ET was performed laparoscopically into one oviduct [69]. Pregnancy was confirmed by ultrasonographic examination on day 21, repeated every 2 – 3 weeks.

### Data description

The analysis is based on data from cloning experiments, performed in the period from April 2008 to February 2011, at the Chair for Molecular Animal Breeding and Biotechnology in Munich, Germany. The location is situated at an altitude of 444 m, and at latitude and longitude of 48°22’N and 11°49’E, respectively.

Changes in the experimental setup, described in the previous section, included variations of the season the ET was performed in, the type of genetic modification, the donor cell source, the number of cloning rounds, and selection of SCNT embryos for development before transfer to the recipient. The stratification and distribution of each varied factor is summarized in Table 2.

1. *Season:* Experiments were performed covering the whole year range, i.e. an approximately balanced sample size in each season – spring (March-May), summer (June-August), autumn (September-November) and winter (December-February) – was ensured. However, 10% more experiments were performed in summer and autumn. The average temperature in each season was 9.6°C, 18°C, 9.2°C, 0.1°C, respectively (http://www.dwd.de).

2. *Type of genetic modification:* Genetically modified cells were derived in roughly 30% of all experiments by additive gene transfer, in 25% of the experiments by homologous recombination, and in most cases (45%) established from transgenic pigs.

3. *Donor cell source:* Regarding the source of nuclear donor cells, most of the experiments were performed with kidney cells (43%), followed by fetal fibroblasts (26%), mesenchymal stem cells (19%), and postnatal fibroblasts (12%).

4. *Number of cloning rounds:* The vast majority of all cloning experiments were carried out with one round of cloning (57%), one third (32%) with two rounds, and the remaining experiments (11%) with three rounds of cloning.

5. *Selection of SCNT embryos for initiation of development:* In 23% of all experiments, all SCNT embryos were transferred to recipients on the same day on which the nuclear transfer was carried out (no selection for development). In other experiments, the SCNT embryos were cultured either 1 day (7%) or 2 days (8%) after activation and selected for initiation of normal development (1-cell stage on day 1, 2-cell to 4-cell stage on day 2). In most of the cases (62%) mixed populations of SCNT embryos (no selection, 1 day culture, 2 days culture) were transferred to the recipients. Those were not included in the analysis of this specific factor.

### Cloning benchmarks

The success of each cloning experiment was progressively assessed based on the outcome of distinct evaluation stages. After the cloned embryos were transferred to the recipient, we first determined whether it became pregnant or not.

For a sample stratum under investigation, the *chance for pregnancy* is hence defined as the probability ratio

(1) $$P\left(\mathrm{pregnancy}=\mathrm{YES}\right)/P\left(\mathrm{pregnancy}=\mathrm{NO}\right)$$

The probabilities result from the relative frequencies of the corresponding event in the stratum.

Analogously, the *chance for delivery* is defined as

(2) $$P\left(\mathrm{delivery}=\mathrm{YES}\right)/P\left(\mathrm{delivery}=\mathrm{NO}\right)$$

For delivering recipients, we counted the number of offspring born, the number of live offspring among them, and if there were any, the number of healthy offspring.

In addition, we calculated for the experiments resulting in at least one delivered offspring the *cloning efficiency* as

(3) deliveredclonedoffspring/SCNTembryostransferred

As a benchmark for oocyte and donor cell quality, respectively, we also took the *oocyte maturation rate,* calculated asand the *fusion rate*, calculated asinto account.

(4) successfullymaturedoocytes/oocytesenteringIVM,

(5) $$\begin{array}{c} \mathrm{successfully}\;\mathrm{fused}\;\mathrm{karyoplast-cytoplast}\;\mathrm{complexes}/\\ \mathrm{complexes}\;\mathrm{submitted}\;\mathrm{to}\;\mathrm{electrofusion}, \end{array}$$

### Statistical analysis

Generalized linear models [70] were computed for each experimental factor (season, genetic modification, cell source, cloning rounds and SCNT embryo selection) in order to estimate its impact on each cloning outcome stage (pregnancy and delivery rate as well as numbers of total, live, and healthy offspring) and the cloning efficiency.

As all explaining variables, i.e. the experimental factors, are categorial, we designed the linear predictor of the regression models using indicator (dummy) variables [71], yielding effects with respect to the correspondingly chosen reference category (*spring* for season, *additive gene transfer* for genetic modification, *mesenchymal stem cells* for cell source, *one round* for cloning rounds, and *no culture* for SCNT embryo selection). This design corresponds to an ANOVA model [72], where the sample mean of each stratum of the experimental factor under investigation is tested for deviation from the sample mean of the reference category assuming the sample means to be *t*-distributed. Consequently, all *p*-values reported here are *t*-test [73] derived, and should, thus, be interpreted as a statistical significance measure for equality of means, i.e. the lower the *p*-value, the more significant is the difference in the means. The link function of the regression models was selected according to the goodness of fit between the empirical distribution of the response (outcome) variable and the corresponding common distribution. Briefly, logistic regression was carried out for the binary factors (pregnancy and delivery), Poisson regression for the counts of live and healthy offspring, and Gaussian regression for the cloning efficiency (as well as for maturation and fusion rate).

## Abbreviations

BAC: Bacterial artificial chromosome; ET: Embryo transfer; IVM: *In vitro* maturation; SCNT: Somatic cell nuclear transfer

## Competing interests

The authors declare that they have no competing interests.

## Authors’ contributions

MK, LG, RZ and EW conceived and designed the study. MK, BK, VZ, NK, AW, AR, AB, KK, KB, KF, TF, CM, TL, MD, AT, SK, DS, HN, AS, EW were involved in somatic cell nuclear transfer experiments. LG, MK, TP, RZ, EW analyzed the data and MK, LG, AK, RZ, EW drafted the manuscript. All authors read and approved the final manuscript.

## Supplementary Material

Additional file 1 **Correlation of the number of embryos transferred with pregnancy rate.** The absolute number of embryo transfers (left *y*-axis) that resulted in pregnancy of the recipient depending on the number of embryos transferred (*x*-axis) is shown in black over the number of all observations in grey. The red curve indicates the overall pregnancy rate (right *y*-axis) when more than *x* embryos have been transferred. Click here for file

Additional file 2 **Correlation of the number of embryos transferred with the number of live piglets.** The number of transferred embryos is shown on the *x*-axis and the number of live piglets on the *y*-axis. No visible correlation can be detected (Pearson correlation 0.2). Click here for file

Additional file 3 **Seasonal distribution of specific SCNT configurations with respect to genetic modification, cell type and cloning round.** For each season on the *x*-axis, the bar height denotes the total number of embryo transfers performed (as indicated on the *y*-axis). The three vertical slots in each of the bars correspond to the distribution of the respective categories of genetic modification (gen.mod), cell type (cell.type), and cloning rounds (clon.rds). The categories are alphanumerically encoded as denoted at the top: genetic modification = (1 = homologous recombination (HR), 2 = additive gene transfer (AGT), 3 = replication of transgenic pigs (replic. of tg pigs)), cell type = (1 = mesenchymal stem cells (MSC), 2 = postnatal fibroblasts (PF), 3 = fetal fibroblasts (FF), and 4 = kidney cells (KC)), cloning rounds = (1 = 1 round, 2 = 2rounds, 3 = 3rounds). Click here for file

Additional file 4 **Distribution of selected embryos derived from specific SCNT configurations with respect to genetic modification, cell type and cloning round.** For a particular selection timing on the *x*-axis, the bar height denotes the total number of embryo transfers performed (as indicated on the *y*-axis). The three vertical slots in each of the bars correspond to the distribution of the respective categories of genetic modification (gen.mod), cell type (cell.type), and cloning rounds (clon.rds). The categories are alphanumerically encoded as denoted at the top: genetic modification = (1 = homologous recombination (HR), 2 = additive gene transfer (AGT), 3 = replication of transgenic pigs (replic. of tg pigs)), cell type = (1 = mesenchymal stem cells (MSC), 2 = postnatal fibroblasts (PF), 3 = fetal fibroblasts (FF), and 4 = kidney cells (KC)), cloning rounds = (1 = 1 round, 2 = 2 rounds, 3 = 3 rounds). Data for mixed selection timing not shown. Click here for file

Additional file 5 **Degree of variation in cloning efficiency within cell types.** The variation in cloning efficiency on the *y*-axis is shown for the different cell lines within the four cell type categories (MSC: mesenchymal stem cells, FF: fetal fibroblasts, PF: postnatal fibroblasts, and KC: kidney cells). The numbers in brackets on the *x*-axis denote the number of embryo transfers (in total and for the corresponding fraction that delivered offspring, respectively). Details on the cell lines used can be found in Additional file 6 and Additional file 7. Click here for file

Additional file 6 **List of *de novo *modified cell lines by additive gene transfer or homologous recombination.** Click here for file

Additional file 7 List of transgenic cell lines from already existing transgenic pig. Click here for file
