# Supplementary figures and images for: Factors influencing the efficiency of generating genetically engineered pigs by nuclear transfer: multi-factorial analysis of a large data set
Source: BMC Biotechnol. 2013 May 20;13:43. doi: 10.1186/1472-6750-13-43 (PMC3691671; doi:10.1186/1472-6750-13-43)

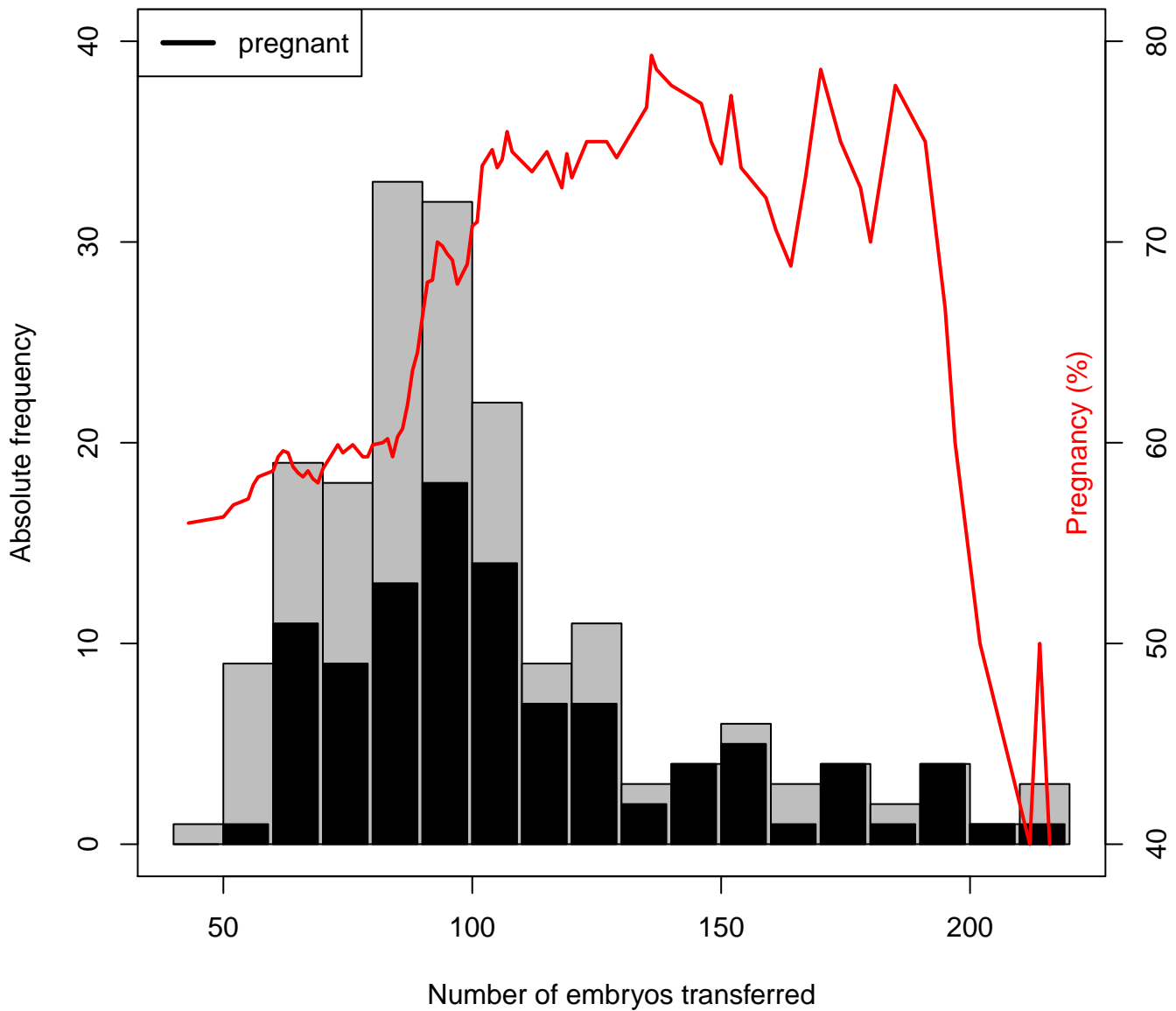

Supplement: Additional file 1 — Correlation of the number of embryos transferred with pregnancy rate. The absolute number of embryo transfers (left y-axis) that resulted in pregnancy of the recipient depending on the number of embryos transferred (x-axis) is shown in black over the number of all observations in grey. The red curve indicates the overall pregnancy rate (right y-axis) when more than x embryos have been transferred. [file 1472-6750-13-43-S1.pdf]

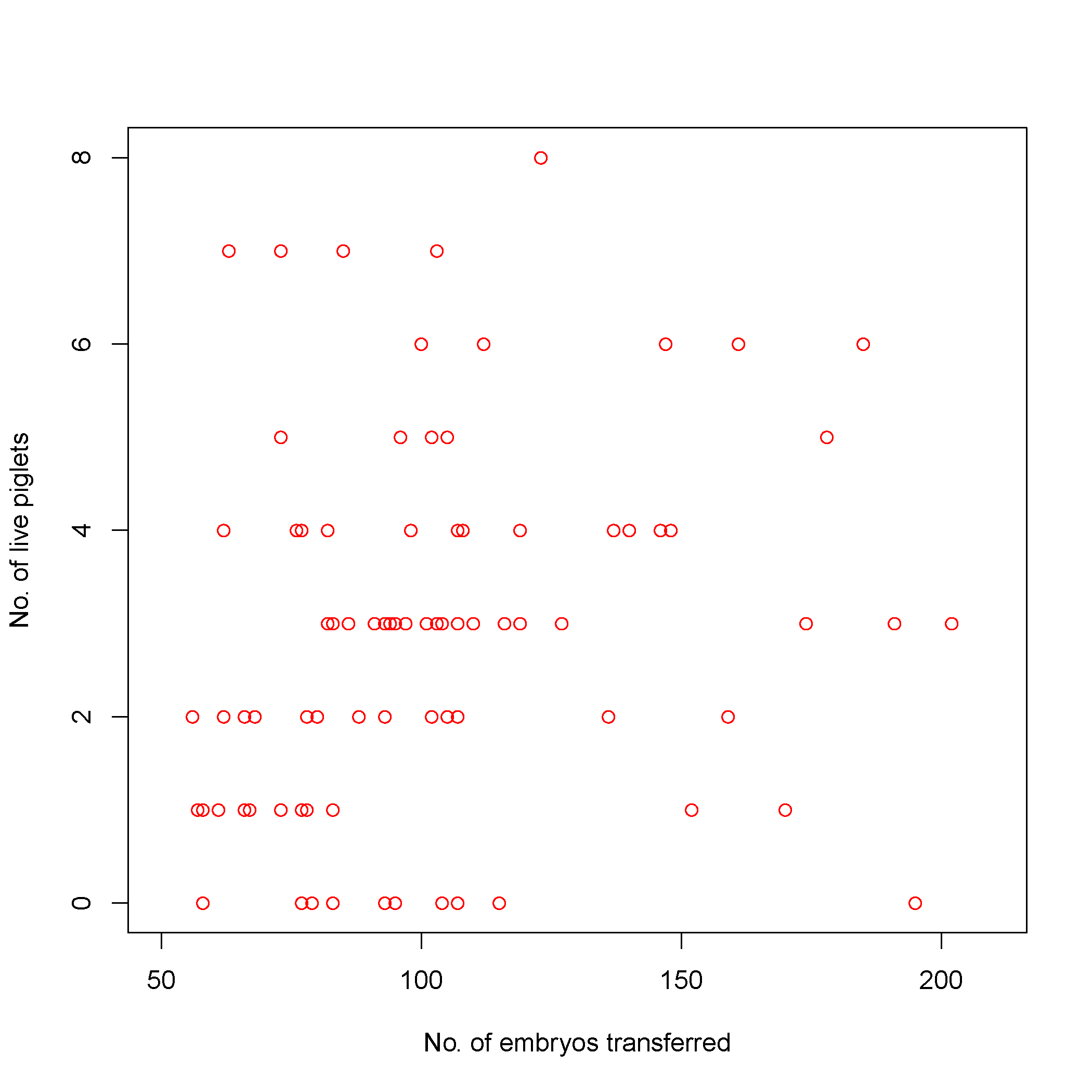

Supplement: Additional file 2 — Correlation of the number of embryos transferred with the number of live piglets. The number of transferred embryos is shown on the x-axis and the number of live piglets on the y-axis. No visible correlation can be detected (Pearson correlation 0.2). [file 1472-6750-13-43-S2.tiff]

gen.mod = (1=HR, 2=AGT, 3=replic. of tg pigs)

cell.type = (1=MSC, 2=PF, 3=FF, 4=KC)

clon.rds = (1=1round, 2=2rounds, 3=3rounds)

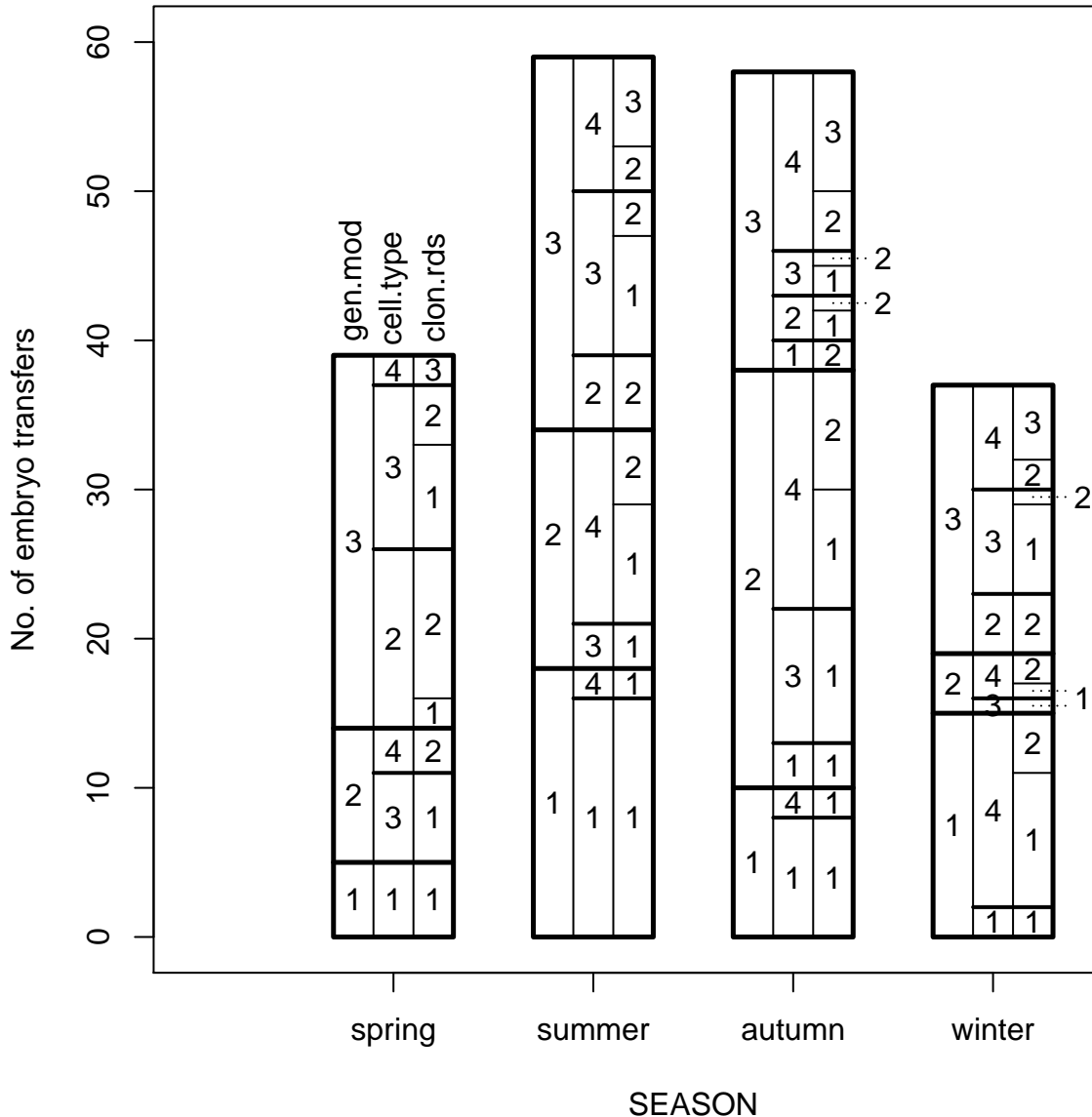

Supplement: Additional file 3 — Seasonal distribution of specific SCNT configurations with respect to genetic modification, cell type and cloning round. For each season on the x-axis, the bar height denotes the total number of embryo transfers performed (as indicated on the y-axis). The three vertical slots in each of the bars correspond to the distribution of the respective categories of genetic modification (gen.mod), cell type (cell.type), and cloning rounds (clon.rds). The categories are alphanumerically encoded as denoted at the top: genetic modification = (1 = homologous recombination (HR), 2 = additive gene transfer (AGT), 3 = replication of transgenic pigs (replic. of tg pigs)), cell type = (1 = mesenchymal stem cells (MSC), 2 = postnatal fibroblasts (PF), 3 = fetal fibroblasts (FF), and 4 = kidney cells (KC)), cloning rounds = (1 = 1 round, 2 = 2rounds, 3 = 3rounds). [file 1472-6750-13-43-S3.pdf]

gen.mod = (1=HR, 2=AGT, 3=replic. of tg pigs)

cell.type = (1=MSC, 2=PF, 3=FF, 4=KC)

clon.rds = (1=1round, 2=2rounds, 3=3rounds)

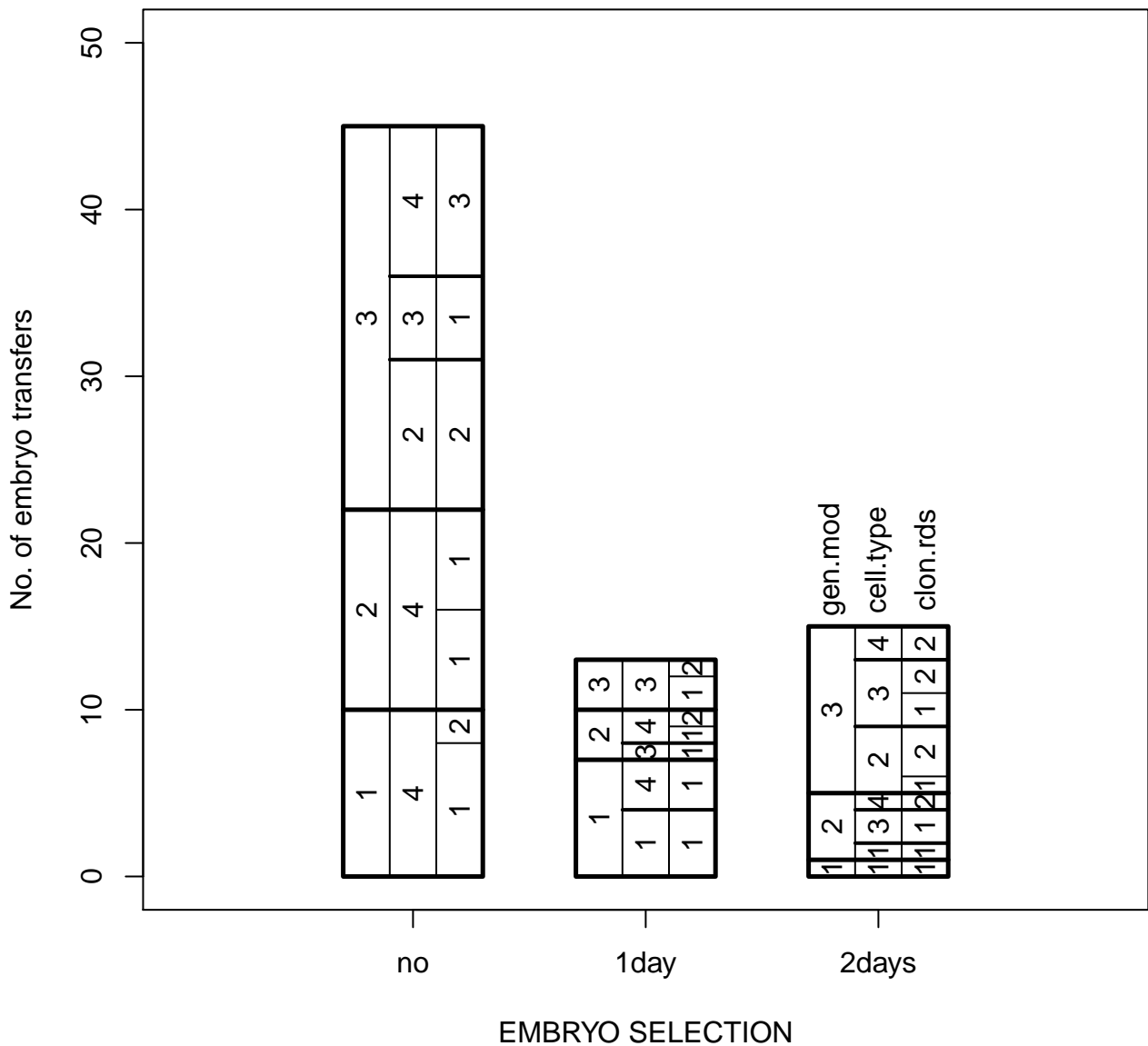

Supplement: Additional file 4 — Distribution of selected embryos derived from specific SCNT configurations with respect to genetic modification, cell type and cloning round. For a particular selection timing on the x-axis, the bar height denotes the total number of embryo transfers performed (as indicated on the y-axis). The three vertical slots in each of the bars correspond to the distribution of the respective categories of genetic modification (gen.mod), cell type (cell.type), and cloning rounds (clon.rds). The categories are alphanumerically encoded as denoted at the top: genetic modification = (1 = homologous recombination (HR), 2 = additive gene transfer (AGT), 3 = replication of transgenic pigs (replic. of tg pigs)), cell type = (1 = mesenchymal stem cells (MSC), 2 = postnatal fibroblasts (PF), 3 = fetal fibroblasts (FF), and 4 = kidney cells (KC)), cloning rounds = (1 = 1 round, 2 = 2 rounds, 3 = 3 rounds). Data for mixed selection timing not shown. [file 1472-6750-13-43-S4.pdf]

Cloning efficiency (%)

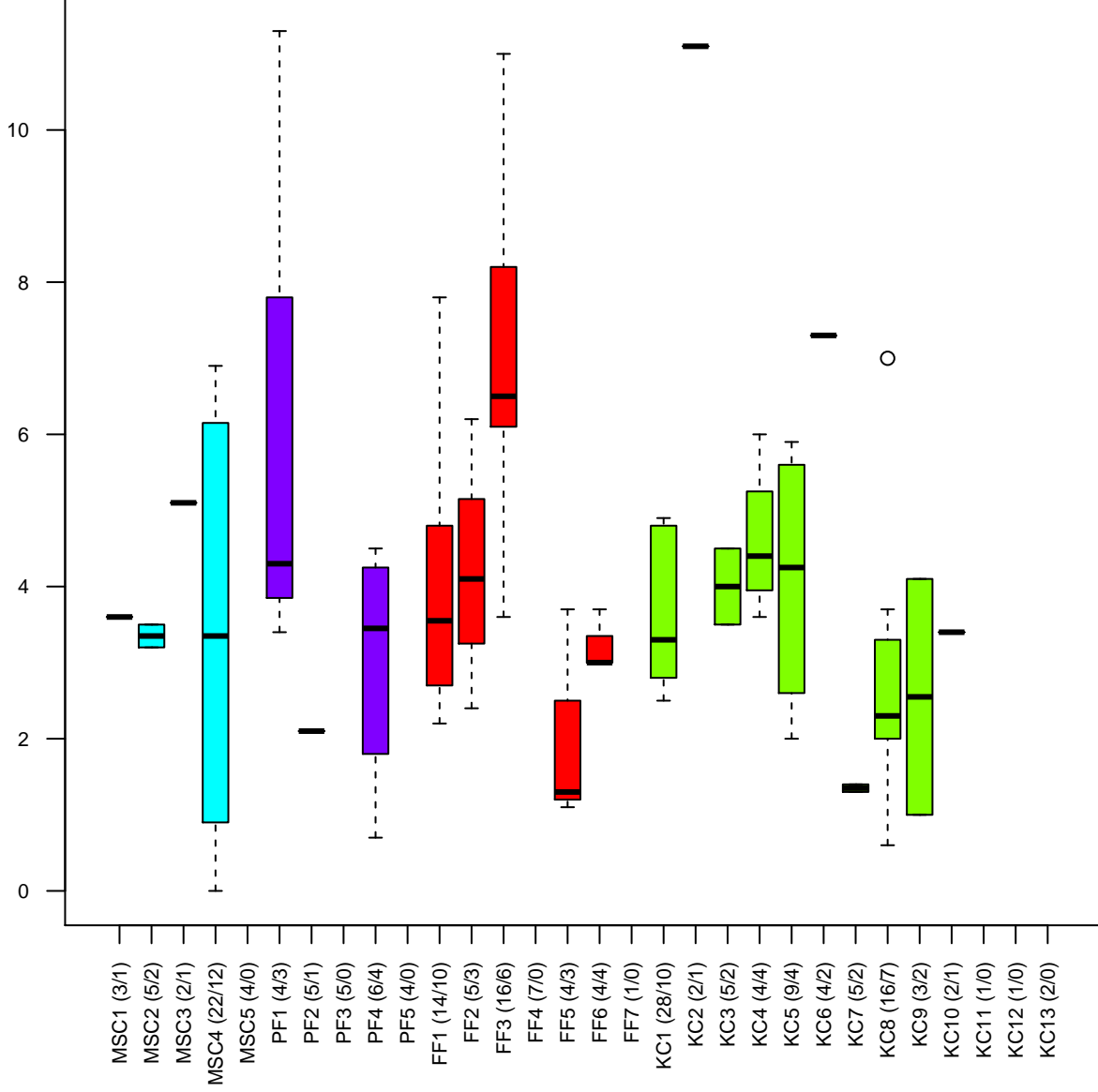

Supplement: Additional file 5 — Degree of variation in cloning efficiency within cell types. The variation in cloning efficiency on the y-axis is shown for the different cell lines within the four cell type categories (MSC: mesenchymal stem cells, FF: fetal fibroblasts, PF: postnatal fibroblasts, and KC: kidney cells). The numbers in brackets on the x-axis denote the number of embryo transfers (in total and for the corresponding fraction that delivered offspring, respectively). Details on the cell lines used can be found in Additional file 6 and Additional file 7. [file 1472-6750-13-43-S5.pdf]
