# Supplementary material for: Factors influencing the efficiency of generating genetically engineered pigs by nuclear transfer: multi-factorial analysis of a large data set
Source: BMC Biotechnol. 2013 May 20;13:43. doi: 10.1186/1472-6750-13-43 (PMC3691671; doi:10.1186/1472-6750-13-43)
Supplement: Additional file 6 — List of de novo modified cell lines by additive gene transfer or homologous recombination. [file 1472-6750-13-43-S6.doc]

**Additional file 6** List of *de novo* modified cell lines by additive gene transfer or homologous recombination

| Genetic modification^1^ | Background of transfected cell^2^ | Cloning round | Selection | Cell line^3^ | Gene construct | Reference |
| --- | --- | --- | --- | --- | --- | --- |
| AGT | WT | 1 | blasticidin | MSC1 | HAC1 | [63] |
|  |  |  | neomycin | FF1 | hTM |  |
|  |  |  |  |  | CAG-Case12 |  |
|  |  |  |  |  | INS-LEA | [62] |
|  |  |  |  |  | INS-C94Y | [16] |
|  |  |  |  |  | INS-C93S |  |
|  |  |  |  | FF2 | INS-C93S |  |
|  |  |  |  |  | CAG-TA | [8] |
|  |  |  |  |  | CAG-LEA |  |
|  |  |  |  | KC1 | INS-TK |  |
|  |  |  |  |  | CFTR-LacZ |  |
|  |  |  |  |  | GGTA-LacZ |  |
|  |  |  |  | KC2 | COL-TK |  |
|  | GM (*GGTA1^-/-^*/ CD46) | 2 | blasticidin | KC3 | hTM |  |
|  | GM (CAG-TA) |  |  | KC4 | TRE-RANKL | [8] |
|  |  |  |  | KC5 | TRE-RANKL | [8] |
|  |  |  |  |  | TRE-CTLA-4Ig | [8] |
| HR | WT | 1 |  | MSC2 | *APC* | [64] |
|  |  |  |  | MSC4 | *APC* | [64] |
|  |  |  |  |  | *KRAS* |  |
|  |  |  |  |  | *JAC3* |  |
|  |  |  |  | MSC5 | *APC* | [64] |
|  |  |  | neomycin | KC1 | *CFTR* | [7] |
|  |  |  |  |  | *GGTA* |  |
|  |  |  |  |  | *DMD* | [42] |
|  | GM (*CFTR^+/-^*) | 2 | blasticidin | KC6 | *CFTR* | [7] |

^1^ AGT: additive gene transfer, HR: homologous recombination

^2^ WT: wild type, GM: genetically modified

^3^ Cell lines isolated from different animals and different tissues (FF: fetal fibroblasts, KC: kidney cells, and MSC: mesenchymal stem cells)
