# Supplementary material for: Factors influencing the efficiency of generating genetically engineered pigs by nuclear transfer: multi-factorial analysis of a large data set
Source: BMC Biotechnol. 2013 May 20;13:43. doi: 10.1186/1472-6750-13-43 (PMC3691671; doi:10.1186/1472-6750-13-43)
Supplement: Additional file 7 — List of transgenic cell lines from already existing transgenic pig. [file 1472-6750-13-43-S7.doc]

**Additional file 7** List of transgenic cell lines from already existing transgenic pig

| Cell line^A^ | Cloning round | Transgene |
| --- | --- | --- |
| MSC3 | 2 | *APC ^64§^* |
| FF3 | 1 | *GGTA1*^-/-^/ CD46 |
| FF4 |  |  |
| FF5 | 2 | *KRAS* |
| FF6 |  |  |
| FF7 |  | *APC* ^64§^ |
| PF1 | 1 | CD46 |
| PF2 | 2 | hTM |
| PF3 |  | INS-LEA ^62§^ |
| PF4 |  |  |
| PF5 | 2 | INS-C94Y ^16§^ |
| KC7 |  | hTM |
| KC8 | 3 | *GGTA1* ^-/-^/ CD46 / hTM |
| KC9 |  |  |
| KC10 |  | CAG-TA / TRE-RANKL ^8§^ |
| KC11 | 2 | *KRAS* |
| KC12 |  |  |
| KC13 |  | *APC* ^64§^ |

^A^ Cell lines isolated from different animals and different tissues (MSC: mesenchymal stem cells, FF: fetal fibroblasts, PF: postnatal fibroblasts, and KC: kidney cells)

^§^ See indicated references
